# Shearwave elastographic evaluation of uterine leiomyomas after uterine artery embolization: preliminary results

**DOI:** 10.3906/sag-1908-171

**Published:** 2020-04-09

**Authors:** Cesur SAMANCI, Yılmaz ÖNAL

**Affiliations:** 1 Department of Radiology, Haydarpaşa Sultan Abdülhamidhan Training and Research Hospital, İstanbul Turkey; 2 Department of Radiology, Fatih Sultan Mehmet Training and Research Hospital, İstanbul Turkey

**Keywords:** Sonoelastography, uterin artery embolization, uterine leiomyoma

## Abstract

**Background/aim:**

We aimed to investigate the role of Shearwave Elastography (SWE) in the evaluation of response to uterine artery embolization (UAE) in patients with uterine leiomyomas.

**Materials and methods:**

SWE images of the dominant uterin leiomyomas were obtained before and 1.5 months after performing UAE in 33 women suffering from symptoms due to leiomyomas (menometrorrhagia, bulk related symptoms, pelvic pain). Leiomyomas were also evaluated by 2 observers for location and longest diameter in axial plane. Interobserver agreement in the quantitative SWE analysis was calculated using intraclass correlation coefficients.

**Results:**

Thirty-three women (mean age, 39.7 years; range, 31–48 years) were examined with SWE 1.5 months after UAE. After treatment, 3 patients (9.1%) had fever, 1 patient had nausea and 29 patients (87.9%) had no complications. The post UAE stiffness measurements of leiomyomas (mean SWE ± SD = 13.34 ± 3.9kPa) were significantly lower than the pre UAE measurements (mean SWE ± SD = 17.16 ± 4.8kPa) (P < 0.001). There was excellent agreement between the 2 blinded observers in SWE measurements.

**Conclusion:**

SWE values of leiomyomas after UAE significantly decreased. SWE, with its high reproducibility, could become a useful tool in the follow up of uterin leiomyomas after UAE.

## 1. Introduction

Uterine leiomyomas are the most common benign tumor in the female pelvis affecting 20%–50% of women [1]. The traditional treatment of uterine leiomyomas is hysterectomy. About 1 in 3 women in the United States is undergoing hysterectomy at the age of 60 [2]. Approximately 67% of hysterectomies in middle aged women are performed due to uterine leiomyomas [3]. Although myomectomy is a less radical surgical technique than hysterectomy, the recurrence of symptoms after myomectomy (15–25% of cases) limits the use of this technique [4,5]. Previously, uterine artery embolization (UAE) was initiated prior to myomectomy [5], later UAE was used as a treatment option for uterine leiomyomas alone [6–13]. UAE is gaining popularity because it is a safe, less invasive and cost effective way of treating uterin leiomyomas causing bleeding, pain, pressure, and mass effect [5–7,14,15].

In previous studies [16–20] ultrasonography (US) and MRI were used to evaluate leiomyomas after UAE treatment. Real-time Shearwave elastography (SWE) is a new, highly reproducible, noninvasive imaging tool [21] that measures the propagation speed of shear waves within the tissue to locally quantify its stiffness quantitatively. 

We aimed to measure uterin leiomyoma stiffness by quantitative SWE before and after UAE and determine whether SWE can be used in the evaluation of leiomyomas after UAE.

## 2. Materials and methods 

### 2.1. Study population and indications 

The Local Ethics Committee of the Fatih Sultan Mehmet Training and Research Hospital approved the study and written informed consent was obtained from all participants before inclusion in the study. Thirty-three premenopausal women suffering from menometrorrhagia, bulk-related symptoms, pelvic pain due to leiomyomas treated by selective UAE were enrolled in this study. All patients were informed of the possible risks and expected benefits of the treatment and written informed consent was obtained from the patients. The mean age of the patients was 39.7 years (range, 31–48). Transabdominal gray scale US was performed to the pelvic region before all patients were included in the study. All patients also had pre- and postoperative contrast enhanced MRI. The purpose of these imaging studies (US and MRI) was to determine the location, number, and size of the uterine leiomyomas and if there was any accompanying pathology (adenomyosis, hydrosalpinx, malignancy), and identify possible complications after the procedure. The patient was not included in the study if there was pedunculated subserosal leiomyoma (because tumor may separate from the uterus and drift into the peritoneal cavity after UAE). In addition, when submucosal leiomyomas were pedunculated they were not included in the study because they could break down to the endometrial cavity and cause sepsis after cervical obstruction. If the submucosal leiomyoma was single and appropriate for hysterescopic resection, the patient was directed to resection. Also, if there is any clinical finding of an infection in the genital tract, leiomyoma is asymptomatic, or if there is a suspicion of pregnancy, the patient was not included in the study. 

### 2.2. Sonography and elastography technique

All US and SWE examinations were performed with a convex 6 MHz frequency transducer (Toshiba, Aplio 500 Platinium, Japan). Transabdominal SWE measurements of the leiomyomas were performed by 2 radiologists after measuring the dimensions of the leiomyoma by transabdominal gray scale US to the pelvic region before embolization. These measurements were performed a few days before UAE treatment and after an average of 44.2 days (39–54 days). Patients were taken after at least 4 h of fasting. Even though more than 1 leiomyoma was present in a patient, measurements and statistics were performed on the dominant uterine leiomyoma. Both of the radiologists were blinded to each other. The interobserver agreement between these 2 radiologists was calculated. All SWE measurements were performed while the patient was lying in a supine position. To prevent the compression effect of the probe, the operator placed the transducer onto the skin surface and kept the transducer stationary during the procedure. Measurements on elastography were performed from transverse US images. The software displayed both speed and propagation maps at dual mode. The software also automatically provided an electronic box which displayed tissue stiffness in a chromatic scale with progression from blue to red, indicating low to high shear modulus (stiffness). The integrated SWE software allowed for placement of circular regions of interest (ROIs) within the elastography window, and automatically displayed shear modulus data (in kilopascals, kPa) for the ROIs. Each radiologist performed 3 measurements with 2 mm ROIs at approximately the same depth through the electronic box and these 3 ROIs were averaged. 

### 2.3. UAE procedure 

Except 3 patients, all patients underwent bilateral UAE under fluoroscopic control by using a biplane angiographic system (Artis zee Biplane; Siemens, Erlangen, Germany). Unilateral embolization was performed in 3 patients because one of the uterine arteries could not be catheterized. All procedures were performed with local anesthesia (prilocaine hydrochloride 1%, 10 cc). Paracetamol infusion was performed prophylactically for pain palliation. Bead Block (Bead Block; Biocompatibles, Farnham, England) 500–700 μm was used in all patients for embolization. Embolizing agent were suspended in 6–7 mL of normal saline and 6–7 mL of nonionic contrast material. Stagnant flow with a pruned tree appearance and no residual hypervascularization related to the leiomyomas were the angiogenic end points for embolization (Figure). There were no significant anastomoses between the uterine and ovarian arteries, so no procedures were canceled. 

**Figure 1:**
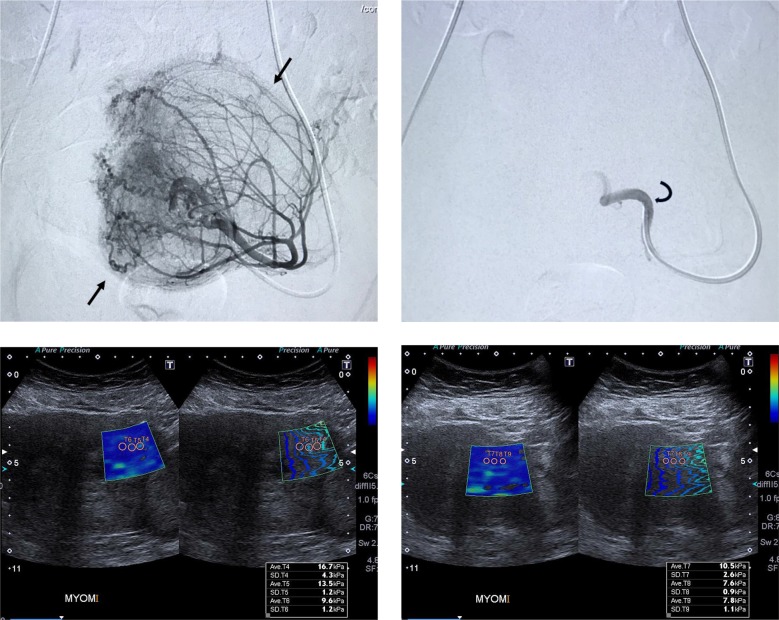
Uterine arteriogram obtained from 43-year-old woman with an uterine leiomyoma. Arrows outline the blood vessel supply of the leiomyoma. b. View of the same uterine artery after uterine leiomyoma embolization. No distal flow is noted. (Curved arrow points to catheter tip.) Ultrasound and shearwave elastography (SWE) images of the leiomyoma. c. before and d. 1.5 months after embolization. There is a significant decrease in the SWE measurements of the leiomyoma after treatment. The vertical bar on the right shows the elasticity range, the change in colors inside the box from dark blue to red indicates increasing stiffness.

### 2.4. Postembolization management and pain evaluation

All patients were hospitalized in the hospital on the evening of the procedure. A 1 mg loading dose of morphine was given to each patient before UAE. During and after the procedure, 1 mg morphine was delivered every 7 min as required, with a maximum dose of 12–15 mg every 4 h. Nonsteroidal antiinflammatory drugs were also given to patients intravenously during and after the procedure. Approximately 5–6 h after the procedure, the patients were asked to select one of the options: no pain, moderate, or intense pain by asking their verbal scale of pain. All patients except for 2 were kept under control for 24 h. One patient stayed for 12 h because she wanted to go to home at night, and 1 patient was discharged after a total of 48 h in the hospital because her pain was persistent. When patients were discharged, nonsteroidal antiinflammatory drugs were prescribed and they were advised to use them if they experienced pain.

### 2.5. Statistical analysis

Statistical analyses were performed using the SPSS software (version 16.0, SPSS Inc., Chicago, IL, USA). Quantitative variables were expressed as mean ± standard deviation and categorical variables as frequencies or percentages. The mean elasticity and mean standard deviation within the ROI are expressed as the mean of 3 different measurements. Baseline data were evaluated using the Shapiro–Wilk test, which showed that the elastography values were normally distributed. Paired sample t tests were performed between follow up, and the baseline reference SWE values assessed differences in elastographic data. Statistical significance was accepted at a P-value of less than 0.05. Interobserver agreement in the quantitative analysis was calculated using intraclass correlation coefficients (ICCs) from a one way random effects model analysis of variance, with the subject as the random effect. A 95% confidence interval (CI) was constructed for each ICC. An ICC greater than 0.80 indicated excellent agreement. Correlations between variables on patients were evaluated by Pearson’s correlation coefficient value.

## 3. Results 

There was 1 leiomyoma in 17 patients (51.5%), 2 leiomyomas in 15 patients (45.5%), and 3 leiomyomas in 1 patient (3%). All of the patients were premenopausal. The largest leiomyoma of each patient was included in the study. Of these dominant myomas, 15 (45.5%) were subserose and 18 (54.5%) were intramural. 28 patients (84.8%) had menorrhagia and 30 patients (90%) had bleeding, pain, and pressure. A mean of 2.7 bottles (minimum 1, maximum 6 bottles) of microspheres were used during the UAE procedure. 

After UAE, 3 patients (9.1%) had fever, 1 patient had nausea, and 29 patients (87.9%) had no complications. Two patients with fever were treated with antibiotics. No procedure was canceled due to a complication. All patients except 3 were bilaterally embolized. In 3 patients (9.1%) one of the uterine arteries could not be catheterized. There was no uteroovarian anastomosis in any patient.

Patients who underwent bilateral UAE were compared to those with unilateral UAE with student t test. Although the rate of decrease in stiffness of the leiomyomas was higher in bilateral embolized patients than that of the unilateral ones (mean + SD; 22% ± 12 and 13% ± 0.3), this difference was not statistically significant (P = 0.253). After the procedure, 90.9% of the patients recovered from the complaints and 3 patients (9.9%) did not fully recover from bleeding, pain, and pressure.

In Pearson’s correlation analyses preoperative leiomyoma size, vials of PVA used (r = 0.87; P < 0.001), and postembolization pain (r = 0.62; P < 0.01) were significantly correlated. Also technical success (bilateral UAE) was significantly correlated with relief from symptoms (r = 0.63; P < 0.001) and menstrual bleeding changes (r = 0.61; P < 0.001). Menstruel bleeding changes after treatment are shown in Table 1. 

**Table 1:** Menstrual bleeding changes.

Change category	1.5 months
Complete resolution	25 (75.8%)
Significant improvement	6 (18.2%)
Slight improvement	1 (3%)
No change	1 (3%)

Approximately 5–6 h after the treatment, patients were asked about their pain verbally; 3 patients (9.1%) experienced intense pain, 14 patients (42.4%) had moderate pain, and 16 patients (48.5%) had no pain after the procedure. No reintervention was made for UAE in any patient.

The longest transverse dimensions of the leiomyomas measured by US were 53.48 ± 15.2mm (range: 35–119) before the procedure and 41.45 ± 12.2mm (range: 22–88) 1.5 months after the procedure. Compared with the paired sample t test, leiomyoma size was reduced statistically significantly (P < 0.001) (Table 2). 

**Table 2:** Mean SWE values and longest diameters of uterin leiomyomas before UAE and 1.5 months after UAE.

	Before treatment(n = 33)	After treatment(n = 33)	P-value
SWE (mean ± SD)	17.16 ± 4.8 kPa	13.34 ± 3.9 kPa	<0.001*
Longest diameter in axial plane	53.48 ± 15.2 mm	41.45 ± 12.2 mm	<0.001*

SWE: Shearwave elastography *Statistical comparison between gastric malignancies and benign gastric pathologies with paired sample t-test.

33 dominant leiomyomas in 33 patients were evaluated by SWE with 2 different radiologists a few days before UAE and an average of 44.2 days after the procedure (39–54 days). The post UAE stiffness measurements of leiomyomas (mean SWE ± SD = 13.34 ± 3.9kPa) were significantly lower than the pre-UAE measurements (mean SWE ± SD = 17.16 ± 4.8kPa) (P < 0.001) (Table 2). Figure shows representative images of pre-UAE angiography, the UAE procedure, and the pre- and post-UAE SWE examinations. Interobserver variability results are shown in Table 3. There was excellent agreement between the 2 blinded observers in SWE measurements. 

**Table 3:** Interobserver variability for SWE measurements.

		Reader 1(mean ± SD)	Reader 2(mean ± SD)	ICC intraclass correlation coefficient
Mean SWE	Pretreatment SWE values (n =33)	17.16 ± 4.8 kPa	17.03 ± 4.7 kPa	0.988 (0.976–0.994)
	Posttreatment SWE values (n = 33)	13.34 ± 3.9 kPa	13.64 ± 3.6 kPa	0.976 (0.952–0.988)

SWE: Shearwave elastography, ICC: Intraclass correlation coefficients, SD: standard deviation.

## 4. Discussion

UAE is a new generation safe and cost effective treatment method which has been used in the treatment of uterin leiomyomas in recent years [6,22,23]. The aim of this treatment is not only to reduce the size of the leiomyomas but also to eliminate the pressure effect, reduce vascularity, and relieve the patient’s complaints [20]. 

Since the principle of treatment is to occlude micro-level vessels with particles, when evaluating the treatment, it may not be enough to say that only the size of the leiomyomas is reduced because the patient’s complaints can be dramatically improved, although the size of the leiomyoma does not decrease significantly [19]. In our clinical practice, some patients showed dramatic improvement in their complaints such as pain and bleeding even though the leiomyoma size did not shrink much. Therefore, instead of evaluating only the size or vascularity of leiomyomas, we designed this study by planning to evaluate the mechanical structure of leiomyomas. Studies using US, Doppler US, and MRI have already been published about patients undergoing UAE therapy [15,17,19,24,25]. US and Doppler US allow evaluation of the size and echogenicity of the leiomyomas as well as vascularization. MRI examination shows the pelvic organs, leiomyoma and uterus perfectly, but it is quite costly especially when gadolinium is used [26].

SWE is a noninvasive and real-time imaging method that measures the stiffness of the tissue. SWE has been popular recently and has been used in many areas, mainly thyroid and breast diseases [27–29]. The most important advantage of SWE is that it is a quantitative, noninvasive method that gives values in kpa [30]. Our aim was to evaluate the feasibility and reproducibility of SWE for showing the effects of UAE on the leiomyomas and assess the value of SWE measurements as a new diagnostic approach for evaluation of mechanical structure of leiomyomas and change of tissue stiffness after uterin leiomyoma treatment. 

As we mentioned with more detail in the results section, in our study, posttreatment elastography values significantly decreased in patients after UAE compared to pretreatment values (table 2). In order to understand the pathophysiology of this decrease, it would be useful to examine the studies on leiomyomas in which surgical specimens were examined by the pathology after UAE was performed. 

McLucas et al. [31] examined leiomyomas pathologically after UAE treatment with 500–700 mm particles similar to ours. They observed diffuse ischemic (coagulative) necrosis with interstitial edema which causes separation of smooth muscle fibers and expansion of interstitial compartment. Ischemic changes were characterised by a loss of viability and architectural detail. PVA particles occluded the vein, a foreign body giant cell reaction developed against this synthetic material and necrosis developed with this mechanism. 

 In a study by Cornelis et al. [32], in a patient, ischaemic necrosis of the leiomyoma was seen 1 month after UAE. They observed a hyaline necrosis of the arterial wall in an ischaemic uterin leiomyoma surrounded by a chronically inflamed myometrium, with very dilated blood vessels, primarily veins. Subserosal arteries and a small vein were completely occluded by a lattice of embolic particles, associated with an intense foreign body giant cell reaction. As the elastography is a method of measuring the stiffness of the tissue, the decrease in SWE values in the evaluation performed after an average of 1.5 months can be explained by the above-mentioned pathophysiological mechanisms like ischemic (coagulative) necrosis. 

Gordic et al. [33] evaluated hepatocellular carcinomas (HCC) with MR elastography (MRE) after radioembolization or transarterial chemoembolization plus radiofrequency ablation. The results of their preliminary study showed that treated HCCs had significantly lower tumor stiffness compared to untreated ones. Also, Li et al. [34] evaluated MRE derived stiffness in human colorectal carcinoma cells, which were implanted subcutaneously in the flanks of genetically modified mice. MRE was performed before and 24 h after treatment with either the vascular disrupting agent ZD6126 (N–acetylcolchi– nol–O–phosphate) or vehicle control. No significant changes were observed in the vehicle-treated cohort. However, ZD6126 induced a significant decrease in tumor stiffness and this was associated with histologically confirmed central necrosis. These results are similar to our study and show that histological cell death and reduction in cellularity may change the mechanical properties of the tissue and reduce the stiffness of the tissue. We looked at the percentages of decrease in stiffness in SWE after the UAE procedure in patients who said pressure effect decreased and patients who said there was no relief in that symptom. Stiffness reduction rate was 28.5% in the group that stated that the pressure effect decreased or passed, and it was statistically significantly higher than the other group (16.2%). This was probably the result of the reduction in stiffness of the fibroid so less pressure on the bladder due to possible mechanisms such as ischemic (coagulative) necrosis with interstitial edema after UAE mentioned above. The goal of embolization is the ischemic infarction of the fibroid tumor, a result that is verifiable with follow up imaging after embolization [16]. From a technical point of view, failure of embolization occurs when the fibroid tumor is not infarcted. In about 10% of treated patients, no improvement of symptoms, which corresponds to treatment failure, is observed [11].

In an MRI study [17], MRI showed a decrease in leiomyoma size as in our study after 3 months from UAE. In addition, increased signal on T1 weighted MR images and decreased signal on T2 weighted MR images were observed due to blood elements and hemorrhagic infarction. Of course, if the measurements in our study were performed 6 months or 1 year after treatment, different results could be obtained. For example, in a study by Goodwin et al. [7], leiomyoma degeneration was seen histopathologically in patients who underwent hysterectomy for treatment failure at the 7th and 14th months. For this reason, the mid–term and long–term results can be evaluated by new studies conducted with larger patient groups and longer intervals.

When we evaluated the interobserver agreement in SWE measurements, we found that two observers achieved highly compatible results with each other (Table 3). This was very important in terms of demonstrating the reproducibility of this method in the evaluation of leiomyomas. Because SWE is a relatively more studied method in superficial organs than in deeply located organs [27–29]. 

In our study, the average size of leiomyomas showed a statistically significant decrease after UAE as expected. Leiomyoma size was evaluated in different studies in patients treated with UAE; Tranquart et al. [19] observed a 29% decrease in leiomyoma volume at 3 months after UAE, 46% at 6 months, and 55% at 12 months. McLucas et al. [20] reported 36% volume reduction in UAE after 1.5 months, 49% after 6 months, and 52% reduction after 12 months. As the months have passed, the size has gradually decreased. Looking at these studies, we can say that as the follow up period is longer, more size decreases are expected. 

In correlation analyses, leiomyoma size, vials of PVA used, and postembolization pain were significantly correlated. We have interpreted this data as more pain was due to increased vials of the particle used because of the large size of the leiomyoma. Likewise, as can be predicted, there was a positive correlation between the ability to catheterize 2 uterine arteries and the relief of complaints and menstrual bleeding changes.

Although we showed the decrease in elastography values after treatment in leiomyomas, in order to evaluate its clinical reflection, we compared fully recovered menstrual bleeding group with the other groups. The decrease in elastography was significantly higher in the group whose bleeding was completely normalized compared to those whose bleeding did not change or slightly improved (P < 0.05). In other words, this change was evaluated not only as numerically significant but also as a clinical reflection. 

Our study had some limitations. As stated previously, it would be better if several more follow ups were made at longer intervals. We evaluated the dimensions with the widest diameter; it would be better to calculate the volume and make more objective size change analysis. We did not analyze the results of some imaging methods that could have been additionally performed, such as Doppler ultrasonography and MRI. Although this issue is important, the focus of this study was the assessment of SWE for measuring the leiomyoma stiffness. The small number of patients remains another limitation of this study.

In conclusion, our study shows that the stiffness of uterine leiomyomas decreases in SWE measurements performed 1.5 months after UAE. SWE, with its high reproducibility, could become a useful tool in the follow up of uterin leiomyomas after UAE, in conjunction with US and other imaging techniques. More data from more patients and longer follow up times are needed to assess the clinical relevance of our preliminary findings. 

## Acknowledgement/Disclaimers/Conflict of Interest

The authors declare that they have no conflict of interest.

## Informed Consent

The Ethics Committee of the Fatih Sultan Mehmet Training and Research Hospital approved the study and written informed consent was obtained from all participants.
